# Haïti and the health marketplace: the role of the private, informal market in filling the gaps left by the state

**DOI:** 10.1186/s12913-015-1088-5

**Published:** 2015-09-28

**Authors:** J. Durham, Marcos Michael, P. S. Hill, E. Paviignani

**Affiliations:** University of Queensland, Faculty of Medicine & Biomedical Sciences, School of Public Health, Brisbane, Australia

## Abstract

**Background:**

In most societies the health marketplace is pluralistic in character, with a mix of formal and informal providers. In high-income countries, state regulation of the market helps ensure quality and access and mitigate market failures. In the present study, using Haiti as a case study, we explore what happens to the functioning of the pluralistic health marketplace in severely disrupted environments where the informal sector is able to flourish.

**Methods:**

The overall research design was qualitative. Research methods included an extensive documentary and policy analysis, based on peer-reviewed articles, books and “grey” literature--government policy and program reports, unpublished research and evaluations, reviews and reviews from key multilateral and bilateral donors, and non-government organisations, combined with field site visits and in-depth key informant interviews (*N* = 45).

**Results:**

The findings show that state fragility has resulted in a privatised, commoditised and largely unregulated and informal health market. While different market segments can be identified, in reality the boundaries between international/domestic, public/private, for profit/not-for-profit, legal/illegal are hazy and shifting.

**Discussion:**

The lack of state capacity to provide an enabling environment, establish, and enforce its regulatory framework has resulted in a highly segmented, heterogeneous and informal health market. The result is deplorable health indices which are far below regional averages and many other low-income countries.

**Conclusions:**

Working in fragile states with limited capacity to undertake the core function of securing the health of its population requires new and innovative ways of working. This needs longer time-frames, combining incremental top-down and bottom-up strategies which recognize and work with state and civil society, public and private actors, formal and informal institutions, and progressively facilitate changes in the different market functions of supply, demand, regulation and supporting functions.

## Background

Most contemporary health systems are mixed in character. That is, to varying degrees they consist of a mix of public and private healthcare providers, and both bio-medical and traditional practices with a variety of financing mechanisms. In many countries this mix of providers is understood in terms of competing health markets, based on the assumption that competition between providers facilitates improvement in efficiency and consumer choice [1]. For competitive health markets to work effectively however, they need to be regulated through law, regulation, or contracts, and in most high-income countries relatively well-established market regulation mechanisms exist and are enforced [2, 3]. In many low and middle-income countries on the other hand, effective regulatory arrangements are often constrained by limited resources, poor understanding by regulators of their role and low incentives to take action in the case of non-compliance [4]. In such contexts, often those who are wealthier benefit from relatively effective regulatory mechanisms, while the poor typically rely largely on informal, unregulated health markets [5].

Tschumi and Hagan [6] argue that within a market system, there are three main sets of functions: core, rules and supporting functions. The core relates to the central set of exchanges between the supply and demand-side of goods and services. These exchanges are shaped by both formal and informal “rules of the game” and supporting functions. Formal rules are usually the domain of governments or professional bodies. Informal rules on the other hand, are generally a product of local culture and value systems [6]. Supporting functions include consultation processes; research and development; information; co-ordination; and infrastructure [6]. To ensure that health markets function efficiently and equitably, the most critical role of the government is to provide an enabling environment and establish the ‘rules of the game’ for individual markets to function [7]. Understanding how markets work in fragile states, where governance is very weak and health outcomes may be even poorer when compared to other low-income countries, is an important step in developing transitional strategies that may complement limited state capacity [8].

This research, examining the health markets of Haïti as an example, allows us to explore how these core functions within the healthcare market adapt when the state is unable to meet its responsibilities to provide health services to its population. It examines how the health market evolves in response to state fragility, and what rules—formal and informal—develop in the context of limited state governance. The paper discusses how in the absence of state capacity to establish and enforce a regulatory and coordination framework, a highly pluralistic and informal health market has evolved which operates largely outside of the legislative frameworks governing heath care. Further, while we present our findings under the different market segments of domestic, international and pharmaceutical, in this informal health marketplace where regulation is weak, the boundaries between the different segments are often blurred and dynamic making such distinctions somewhat artificial. We argue that in this context the size, scope and reach of public health services are severely reduced, leaving space for the establishment of a health market driven largely by self-interest. We also draw attention to the need for donors, service providers and health services researchers in fragile states to extend their analysis beyond the formal public health sector, which in such contexts, provides a relatively limited amount of healthcare services. While we cannot draw generalizations from a single country case study, we aim to highlight the importance of informal markets in complex and fragile environments and the need for donors and non-state actors to engage with the state despite its limitations.

The challenges of undertaking research in fragile and conflict affected states means the there is a lacuna of knowledge of how health systems work in such environments. Further, much of the health literature related to such environments has focussed primarily on aspects related to Western aid or the public sector even when the state is largely absent. Such a focus however, ignores much of the healthcare arena. This paper is important in adding to our current limited knowledge base of how health markets evolve in response to total or partial state failure. It considers the usually overlooked informal mechanisms that allow for healthcare provision in such difficult environments, and points to the need for donors—and governments—to develop new modes of engagement with these emergent service providers. While recognising the challenges, we also call for further research to deepen our understanding of how to optimise the contribution of these health markets that continue, despite social, political and economic disruption, to deliver health in fragile and conflict affected states.

### Haïti

Haïti’s two centuries of independence have been plagued by poverty, violence, political instability, violent overthrows, successive coups, countercoups and dictatorship, alternating with occupation by foreign troops, including those of the United States of America from 1915 to 1934 [9]. This fragile situation has been further exacerbated by repeated disasters, often of a huge scale, which have diverted both international and domestic resources to humanitarian goals at the expense of development ones. These repeated political and natural disasters result in ‘routinized ruptures’ and a continuous state of ‘insecurity’ or ‘ensekirite’ as it is termed in the local Kreyòl, which undermine Haïti’s efforts to consolidate its democracy and create a climate of peace and security [10].

Haïti is the poorest country in the Western Hemisphere and one of the highest aid-recipient countries in the world. The country’s Human Development Index 0.458 ranked 161 of 179 in 2012, and has remained roughly stagnant since 2005, as opposed to the climbing indices in Latin America and the Caribbean [11]. This extreme poverty reflects on social indicators such as literacy, life expectancy, infant mortality, and child malnutrition [12]. Officially, Haïti’s health system is divided into four sectors: 1) public; 2) private not-for-profit (NGOs and religious organizations); 3) mixed not-for-profit (private management with staff paid by the Ministry of Public Health and Population (MSPP); 4) private for-profit [13]. Overall management of the decentralised health sector resides with the MSPP, with delegated responsibility to the Départements, although its management capacity is weak and public health providers operate with variable population reach and quality [13]. Most primary health care is provided by non-state actors, while the state retains the main responsibility for secondary and tertiary care, most of which is concentrated in Port-au-Prince, with fees for services and products that are often beyond the reach of the majority of the population [14, 15]. The 2011 earthquake further disrupted the already fragile public health system, destroying much of the MSPP central infrastructure, and resulting in a large increase of International NGO health services [16].

The quality of the healthcare system is reflected in its basic health indices, which compare poorly with its regional neighbours: the EMMUS-V survey estimates life expectancy at birth to average 62 years; a maternal mortality ratio of 350 per 100,000 live births compared to a regional ratio of 63, in part explained by only 38 % of births with skilled birth attendants (59.6 % urban, 24.8 % rural) compared with 93 % across the region [17]. Under-5 mortality is 88 deaths per 1000 live births, and DPT3 rates hover around 62.5 % compared to regional averages of over 85 %. Despite comparable TB treatment success rates, prevalence at 314 per 100,000 is multiples of the regional prevalence of 36, and linked inevitably with its HIV at 2.2 % prevalence — the highest in Latin America [12]. Acute malnutrition among under-5 children is 5 %, and chronic malnutrition 22 % [17], and is the highest in Caribbean and Latin America. Infectious diseases continue to cause unnecessary deaths and chronic diseases are increasing [18].

## Methods

This research is one of six country case studies, examining the provision of health services in fragile and conflict affected states, largely funded by the Danish Ministry of Foreign Affairs, and coordinated through the Australian Centre for International and Tropical Health (ACITH), at the University of Queensland. The six countries were selected for the diversity of their social, political and historical evolution: Afghanistan, Central African Republic (CAR), Democratic Republic of Congo (DRC), Haïti, Palestine and Somalia. The overall purpose of this larger research project was to provide greater understanding of the provision of health services in fragile states and to examine the ways in which health systems react, adapt and evolve in response to total or partial state failure, and how national and international stakeholders respond to such challenges. The findings of the study have been published in peer reviewed publications and in detailed country reports [19–21], [www.sph.uq.edu.au], [22]. The objective of this component of the Haïtian case study was to examine how the Haïtian health market has evolved in response to state fragility.

The research was grounded in an extensive documentary and policy review, based on peer-reviewed articles, books and “grey” literature—government policy and program reports, unpublished research and evaluations, and reviews from key multilateral and bilateral donors, and non-government organisations. A search was undertaken using databases including Ovid Medline, Embase, Web of Knowledge, Web of Science and web-based searches of data available in the public domain and hand-searching relevant journals. Searches from 1995, with a particular focus on the period since the United Nations peace-keeping intervention in 2004, were undertaken prior to the interviews in 2011, and updated for this analysis. Key words included: health, health systems, health services, conflict and fragile states. Documents were also collected from people working in fragile states, including those who were interviewed. Qualitative, quantitative and mixed method studies and documents in French and English documents were included.

In-depth interviews were undertaken using a topic guide prepared by the research team for all case-studies and adapted to the specific Haïtian context by authors MJM and PSH, who undertook the interviews between January and February 2011 in Haiti. Qualitative interviews were chosen because of their ability to provide in-depth descriptions of the health system and the experiential perspectives of stakeholders [23, 24]. The topic guide used for the interviews related to the overall objectives of the broader study and included question prompts about health service provision and the impacts of social, economic and political disruption on health services. While topic guide was used, the interviews also allowed participants to talk about health service provision in their own words focussing on the issues that they felt were important. This provided the interviewer the flexibility to follow up and clarify participant ideas and adapt interviews as the study progressed and new insights were gained. This means that as is often the case in qualitative research, there were some differences in the ways in which questions were framed and answered [23, 24].

Key informants were purposively selected, using the organogramme of the MSPP to identify senior administrative staff, and the humanitarian donor coordination list, to identify donor and NGO representatives. These respondents identified further relevant informants, and provided contacts with both the Cuban Brigade and representatives of the Département at Cap Haïtien. A seminar at the Université de Notre Dame de Haïti allowed us to present the outline of our research, and facilitated introductions. A total of forty-five interviews were undertaken in Haïti with key representatives of the MSPP, multilateral and bilateral donor agencies, academic institutions, NGOs and private practitioners, in Port-au-Prince and Cap Haïtien. Identified key-informants were contacted by phone, and interviewed at their work premises by one or both researchers, with notes maintained and edited within 24 h following debriefing between the authors and corroboration of findings. Verbal consent was approved by the University of Queensland Behavioural and Social Sciences Ethical Review Committee on the basis that “informants are senior personnel in national or international agencies, responsible for the health systems issues they will be discussing.” The research proposal and ethics approval were provided to the Director-General of the MSPP and the chair of the MSPP ethics committee on arrival in Haïti. Although the key informants were bilingual, interviews were undertaken in French or English (or in the case of Cuban informants, Spanish) depending on the interviewees’ preference. Notes of the interviews were made during the interview, with direct quotations in the language used, but translated by the interviewers prior to analysis. All interviews were undertaken following reading of the information sheet witnessed by the interviewer(s). The information sheet was provided in English, and discussed and clarified in French where necessary prior to verbal consent to proceed. Table 1 summarises the number of respondents by category and location.

**Table 1:** Summary of participants by category and locations

Participant category	Haiti	
	Location	
	Port-au-Prince	Cap Haïtien
MSPP	5	1
Academic (Haïti)	4	
Haïtian Hospital	2	1
Haïtian NGO	7	
Haïtian Consultant	2	
Cuban Brigade		2
Multilateral Organization	8	1
Bilateral Organization	2	
International NGO	7	1
International Consultant	2	

MJM and PSH initially manually coded the interview data from notes primarily recorded in English. Coded data was grouped into the key themes guided by the broader project research questions and corroborated by the second researcher. Subsequently, the Haitian data was reanalysed by JD and PSH, with a focus on those themes relating to health markets and financing.

Triangulation of the qualitative data was achieved by using different methods (program documentation review, interviews) and interviewing participants from different sectors. In addition, field notes and contact forms were maintained ensuring an audit trail. Further credibility safeguards included the authors manually coding the data into themes and integrating the interview data with the document review. The initial draft of the research report was reviewed by the whole research team, edited and submitted to three independent researchers in Haïti for review. Anonymity and confidentiality of participants were protected by the use of coding and strict security measures, which included storing documents and recordings in a locked cupboard and/or on a password protected computer.

## Results

The findings of this research are presented using the different market segments to structure the analysis. Using a systems approach to the analysis, enables an examination of the interconnectedness and blurring of boundaries between the different market segments, the financial incentives that drive supply and the formal and informal institutional arrangements that exist and influence outcomes. This analysis recognises that the Haïtian health marketplace is in essence mixed, with the different practices, financing mechanisms and market segments found in a contemporary health markets. State fragility, limited regulation and coordination of the system, and the significant contribution of the not-for-profit sector to public services means that the lines between public and private healthcare are highly permeable. Thus, although in presenting the findings, for ease of discussion we use terms such as ‘public’ and ‘private’ and ‘domestic’ and ‘international’, we recognise that this is an unrealistically concrete division. The findings illustrate that despite the state being largely absent, a richness of initiatives have developed by a range of providers.

### The domestic marketplace

The domestic market consists of state and private providers. Publicly delivered services are severely underfunded with limited coverage outside of urban areas. Most of the available budget is used to cover salaries. Salaries are low and can force even public servants and doctors with high responsibilities to combine their job with other functions. Low pay, limited facilities and weak accountability mechanisms contribute to an almost dys functional public health system. Doctors in state health facilities interviewed for this study, reported that medical material and drugs in the General Hospital of Port-au-Prince, are bought by the patients (interns and residents at times created informal cross-subsidies by making well-off patients buy more syringes and intravenous fluids than needed, for instance, and using the surplus for poor patients). Interns and residents also must provide their own diagnostic equipment, such as thermometers and sphygmomanometers. A lack of state funding means that each of the public hospitals seeks complementing income and equipment from external sources and partners (which can lead to the paradoxical situation that a District Hospital with an effective director may offer better services than the Départmental Hospital).

The inability of the MSPP to provide sufficient financing in essence makes the public sector commercial, with healthcare workers selling both health services and products, further blurring the lines between public and private. The presence of a large not-for-profit sector also weakens demand for both public services. The private for-profit health sector is primarily located in Port-au-Prince and consists of physicians, dentists, and other health care specialists [25]. Both government and donor informants indicated that the formal for-profit market is very limited, due in part to the high prices in the face of widespread poverty, the opportunity for the wealthy to use hospital services abroad and competition from the not-for-profit sector. The informal for-profit market addresses much of the popular demand among the poorest, with untrained drug sellers offering medicines of questionable quality in the markets. The presence of so many non-state providers contributes to market flaws. This includes an under-supply of population health services, a relative over-supply of curative services, an over-prescription of pharmaceuticals and asymmetrical knowledge between providers and patients.

Another segment of private sector is the traditional market which is used by a large proportion of Haïtians. Jones et al [25] found that nearly half of the population, particularly in the rural areas, relied on traditional medicine. Informants argued that Haïtians were creative in their capacity to “muddle through”, and recourse to traditional medicines Petit-Frère et al. [26] showed use of home remedies for 71.6 % of symptoms in Petit Goâve, and cite other studies with percentages of traditional auto-medication ranging between 60 % and over 89 %. Another study cited revealed a density of one traditional healer for every 107 inhabitants in Artibonite, compared to 2.7 health professionals for every 10,000 inhabitants. Western medical services are concentrated in major coastal centres of population, with only traditional medicine available in the rural hinterland above an altitude of 500 metres [26]. The traditional health sector usually incurs some monetary or non-monetary out of pocket expenses.

### The international marketplace

Interviews with our informants and the literature suggest that the domestic health market—formal and informal, public and private, not-for-profit and for-profit—is integrally linked to a larger, complex international health market. The international not-for-profit segment dominates the healthcare market with the major health spending made through multi and bilateral lateral funding mechanisms, philanthropic funding, public-private partnerships and donor contracting of not-for-profit service providers. The U.S. and Canada are among Haïti’s largest bilateral donors with much of their assistance channelled through non-governmental agencies. It is estimated that before the 2010 earthquake, approximately half of all spending on health services was provided by donors and that between 2009-2010 US$ 333.71 million of overseas development assistance (ODA) was spent on health [27]. The Global Fund for AIDS, Tuberculosis, and Malaria (Global Fund) has provided finding to Haïti since 2003 with country has receiving a cumulative total of US$ 274,991,502 between 2003 and 2014 [28]. Through PEPFAR, Haiti has received $ 773.8 million between 2004 and 2011 [29]. Table 2 shows the largest sources of ODA disbursements for health for the period 2009-2010.

**Table 2:** Five main sources of ODA for health to Haiti 2009-2010^a^

Source	Percent
United States	65.3
Global Fund	12.3
Canada	9.6
UNFPA	5.9
Inter America Development Bank, special fund	2.5

^a^Overseas development assistance: Haiti [http://www.who.int/gho/governance_aid_effectiveness/countries/hti.pdf?ua=1

The 2011 earthquake led to an increase in providers and following the earthquake, there were reportedly more than 400 health organizations and agencies contributing to the response, creating a major coordinating challenge in chaotic circumstances [16]. Emerging health governance was again eroded. One independent Haïtian health consultant described it acutely: “We are not at war, but it is as if we were at war… this instability”. Long term strategic planning is sacrificed to reactive responses, with a senior official from a key multilateral reflecting on the demands of recurrent crises: “things happening all the time… everything is prioritized and urgent, and we don’t manage; it’s a vicious circle.” The subsequent hurricane and cholera epidemics further contributed to significant increases in external donor funding without a corresponding increase in local absorptive capacity; in fact, the past decade has seen what one bilateral agency staff member characterised as “a permanent and inevitable collapse” of the public health sector [30, 31].

Many of the NGOs, especially relief NGOs, are reliant largely on international donor funding and employ a mainly international workforce for management and technical inputs. Respondents explained that the different not-for-profit NGOs work in various ways which include running services in MSPP-owned premises, sometimes with the MSPP providing salaries, or an NGO may pay or top-up MSPP salaries. In addition, there are population level disease-specific, vertical international programs such as Global Fund initiatives for HIV/AIDS, Tuberculosis and Malaria. The MSSP has been successful in several of these collaborations, introduction free obstetric care, reducing HIV prevalence, showing progress in immunization initiatives such as polio. But international organisations have largely assumed control of these vertical programs, despite the coordination mechanisms recently introduced by the MSPP. The massive growth of the international NGO industry — reluctantly tolerated by the state — and the state’s limited available domestic resources, have resulted in a passive stalemate: limited effort has focussed on local capacity building, integration with or transfer to MSPP control.

In addition to NGOs providers, since 1998, the not-for-profit sector has included services through a bilateral agreement: the Cuban Brigade. At the time of the study, the Cuban Brigade worked in 70 health facilities in Haïti for which they were responsible and seconded staff to another 87 health facilities. The policy of the Cuban Brigade is to work alongside local counterparts whenever possible, and to hand over in the long run, to the MSPP although it was reported that there is no fixed timeline for this to occur.

The diaspora was reported to also account for a proportion of national health care expenditures through remittances, provision of services and assistance in linking in-country patients with providers outside of Haïti’s national border. Thus while the international health market dominates the health market place, its links with the domestic market are multiple and complex with the domestic market ultimately dependent on the international market.

### The pharmaceutical market

In the public sector, direct control of the central medical stores was relinquished by MSPP in the 1990s under pressure from international agencies, and a collaborative initiative PROMESS (Programme de Médicaments Essentiels), was established under the management of PAHO. PROMESS is responsible for undertaking needs analysis, creating lists of required drugs and procuring drugs from Washington and other international sources for redistribution to public sector (MSPP and Départements) and registered NGOs with a minimal price margin (about 15-20 %). It also procures other products for MSPP which are funded by donors or other UN agencies (UNFPA, UNICEF) for free distribution in programs in immunization, TB, malaria and filariasis. Increasing regulation around the requirement for registration to access PROMESS benefits however was reported to have led to exclusion of non-registered NGOs from MSPP coordination activities. Nevertheless, NGOs which resist registration and coordination were reported to be able to continue because of their independent supply lines. This has had the unintended consequence of limiting MSPP awareness of sectoral activities to those of compliant, registered agencies.

There are three pharmaceutical laboratories approved to produce pharmaceuticals across a limited range of drugs. Local production however is inadequate, unregulated, and there is no system of quality control applied by the MSPP [25, 32]. Unpredictability of supply and demand that results from the surges of international agency activity, and the direct and indirect consequences of their varied pharmaceutical supply systems, means that local production is unable to be predictably developed. The MSPP now requires importers to be registered and for each drug (by specific brand preparation, not generic active ingredients) importation to be separately authorised. This is perceived by NGOs however, to be both costly and time consuming, with a series of approval steps contributing to delays. Humanitarian relief NGOs, expecting a short time frame of engagement in Haïti, frequently circumvent the requirements--despite expedited processing of approvals being developed by the MSPP. Experienced health consultants working in Haïti also pointed out that “donations” can prove to be very expensive to the MSPP, with the approval processes involving real and opportunity costs for staff within the MSPP. Further, when relief agencies exit, there is often a massive “dump” of residual pharmaceuticals into the market which was reported to be severely disruptive, and persistent, despite strong MSPP requests to ensure alternative exit strategies. The private informal sector was also reported to have its own unregulated supply chains with demand boosted by self-medication, a common practice, particularly among the urban poor. According to several of our respondents, including those in multilateral agencies advising the MSPP, the multiple small scale importers in the private informal sector “escape all attempts at control”. Recourse to private, traditional medicine, particularly in rural areas was reported to be high, where access to modern pharmaceuticals is limited.

### Governance and market regulation

The development of governance and policy within the health sector reflects the broader political history of Haïti, and its “routinized ruptures”. There is a clear link between the fragility of the state and the lack of an effective health system. The capacity of the state to provide health services is constrained, not only by finances and human resources, but by political failures. In the words of one bilateral donor consultant: “the social contract is weak”. He observed that the regular changes in Ministers of Health have further depleted the capacity of the MSPP to develop and introduce policy, as “each time the next starts from a blank slate”. With the loss of corporate memory resulting from the change in the upper bureaucracy that accompanies these political changes, consensus around the underlying values that might guide the development of the health system, is difficult to achieve and the developments from previous policy work are continually undermined. Inefficiencies and duplication are an inevitable consequence, as one Haïtian consultant explained: “it’s a system that doesn’t want to develop, but which nevertheless absorbs a lot of money”. For the donor community, this makes it difficult to align to a “plan” that has limited predictability.

Despite its leadership in state decentralization, as MSPP officials in our sample readily admitted, the MSPP lacks the capacity to implement its decentralization policy and coordinate and monitor the myriad of health actors, including the public sector. Health partners were reported to often start projects without MSPP authorization or with authorization being sought from health authorities subsequent to activities starting. Lack of regulation and often a bypassing of official procedures were reported to create parallel procurement processes which were also vulnerable to corrupt circumvention of the governmental system. The private sector particularly, was reported to be very much beyond the control of the MSPP. In any case, lines of accountability were no longer clear, with one dispirited local consultant reflecting: “there was less impunity; nowadays, a citizen wouldn’t even know where to lodge a complaint”. While the MSPP hosts a ‘Direction de la Pharmacie et de la Médecine Traditionnelle’, traditional health service providers are not regulated, nor is the role of traditional practitioner defined [26]. In addition, health and medical professional training is of uneven quality, suffering from resource constraints and long-standing politicization. About 80 % of graduates are estimated to leave the country, mainly for North America but senior MSPP human resource staff noted that “less than 50 % of these emigrants continue to practice medicine”. Many of those who remain leave the public sector to work in non-state agencies due to better facilities and higher salaries. This institutionally weak position of the MSPP, creates a vacuum in governance and leadership to be filled by the more powerful donor and multilateral organizations. Some of our respondents questioned how this power imbalance affects issues of accountability and whether the plethora of health organizations is accountable to the right groups.

A number of respondents, both within government and the development community, suggested that, given its financial and human resource constraints, the MSPP finds itself in a difficult position: dependent on the not-for-profit sector to provide the service provision that it is unable to assure, while at the same time, uncomfortable with the de facto transition of authority to that sector. A former Minister of Health, on the other hand, accused foreign powers of intentionally weakening the Haïtian state by propping up non-state actors. A number of accusations were also made that MSPP strengthening would actually not be in the interest of private not-for-profit providers such as NGOs and churches. After all, it is not only the stream of funds, but also institutional building and learning that benefit the private not-for-profit, instead of the public sector. Where facilities created previously by an NGO, were handed over to the MSPP, sustainability was cited by many of the respondents as a critical issue, with inadequate planning and accounting for non-deferrable, recurrent expenditure.

Despite the weaknesses of the MSPP, donors, UN agencies and those NGOs with any interest in engaging the MSPP, pay formal lip service to the MSPP as if it were fully in charge, as one Haïtian development practitioner explained, reflecting a view of many of our respondents: “the true leadership lies with the international agencies and their implementers, the NGOs”. Health partners were accused as a result of “acting symbolically and hypocritically” while the MSPP was overwhelmed by the amount of paperwork generated by co-operation projects which need to be approved and reported on. Being entirely donor-dependent, declining proposals was reported as being virtually impossible for the MSPP. A key multilateral advisor to the MSPP observed that “the Ministry doesn’t have the ultimate authority” and that as a result, “the Ministry is always in favour of all proposals”. Yet, pliable as it is to their demands, donors appear to have little confidence in the MSPP. Particularly since the earthquake, the subsequent hurricanes and the cholera epidemic, external donor funding has increased exponentially, without a corresponding increase in local absorptive capacity. Donors now have significant financial investment, and a shared desire to influence change, but there is no consensus between them that would provide a consistent direction to drive change. Despite the substantial financial investment, current initiatives do not build on-going capacity, continuing management structures, or enduring local systems. Indeed, from the perspective of one Haïtian consultant, the current action by many donors to effectively replace state service provision has not produced better results than the state would have done. This directly resulted in “a policy of weakening the state”, but despite undermining state governance, failures were still attributed to local failings: “the argument [made] is always the lack of management, the corruption…”. What results had been achieved quickly perished: “les resultats sont périssables”.

## Discussion

In this paper using Haïti as an example, we have explored through the lens of the marketplace, what happens when the state is unable to comprehensively carry out its key responsibilities in health service delivery across the population. In Haïti, the lack of state capacity to provide an enabling environment, establish, and enforce its regulatory framework and develop a clear vision and plan for the health system has resulted in a highly segmented and informal health market. In this informal health marketplace, there are a plethora of health actors including traditional practitioners and public and private practitioners schooled in professionalized biomedicine. The status of some of these practitioners is not always clear with the boundaries between public/private, for profit/not-for-profit, legal/illegal often hazy and shifting. What is clear however, is that health care beyond the limited reach of state services, has developed into a privatised, commoditised and largely unregulated service. Within this context, as in other fragile states, the scope of public services, including health care provided by the state is extremely limited, yet research has continued to largely focus on this formal public health sector which provides the minority of health care [19–21, 31, 33, 34].

The donor response to this largely dysfunctional health system has been to bridge the supply-side gap with external, non-state, private health providers. In part, this approach has historical roots, with the state devolving responsibility for health services to the not-for-profit sector, in particular the Churches. Essentially, donors through NGOs, public-private partnerships and bilateral agreements have provided the finance, advice, technologies, human resources and services they deem necessary to deliver directed, subsidised health services and products. Alongside this, other private providers have stepped into to fill the vacuum created by state fragility. The presence of these other players acts as an incentive for patients to abandon the public system, thus reducing demand and further shifting stewardship of the health system from the hands of the state to the market. Limited in its capacity to provide adequate services or to coordinate or discipline errant partners, the MSPP often feels unable to refuse any external complement to Haïti’s health services. These factors all contribute to market failure, including counterfeit drugs, inappropriate drug use, inappropriate quality and cost of care and a fragmented health system [5]. As in other fragile or conflict affected states, this condition of fragility and lack of stewardship is not the result of a simple linear process. It is the result of a range of exogenous and endogenous pressures including history, violent conflict, political instability, multiple disasters and human agency. These factors render the state in a perpetuating cycle of fragility and an enduring state of emergency resistant to remedy, vulnerable to recurrent shocks and stuck in underdevelopment [8, 33, 35].

As demonstrated by Haïti’s poor health outcomes, the emphasis on supply-side issues and direct provision of services has yielded limited results. As Barder [36] and Ikpe [33] have observed, failure to focus on demand side issues and institutional capacity building often has the perverse effect of further undermining the states capacity to fulfil its obligations. In Haïti, the strategy of favouring NGOs as service providers has made institution building unsustainable and left issues of accountability and transparency unresolved [35]. Newbrander [34] observes that contracting to NGOs allows for expanding health services quickly, but may substantially bypass government mechanisms, as was suggested in the present study. While it would unfair to attribute the weakness of the Haïtian health system solely to the parallel modus operandi of external actors, failure to engage with the MSPP perpetuates a vicious circle, whose detrimental outcomes impact directly on the Haïtian population. Lewis [37] has also observed that returns on health investments may fail to materialise if institutional capacity building is not addressed. Rather than building up parallel organisations, Cammack et al. [31] call for working through government institutions as much as possible. Where alignment with the host government’s plans and procedures is not possible, they recommend considering ‘shadow systems alignment’, whereby delivery is compatible with existing or future state structures.

The weakness of the MSPP is a reflection of the weakness of the state itself and it is unrealistic to expect the MSPP to initiate change on its own. Further, the non-state actors, for and not-for-profit, appear to be quite content with the status quo. A request for regulation, for respect of standards and accountability is unlikely to come from their side. Donors—multilateral and bilateral—are therefore left as the most likely initiators for change. For donors however, funding non-state actors is a way of much less resistance than trying to work through the public sector which admittedly would not be without risks. Maintenance of the status quo however, carries an enormous deficit in terms of effectiveness, efficiency and accountability and ultimately is not a viable option. Clearly, current donor strategies are not leading to sustainable, improved health outcomes, nor is an imposition of regulatory frameworks and approaches from high-income countries realistic [5]. Thus donors need to look for new, more nuanced, incremental ways to engage with the state in fragile environments where informal markets prevail [18, 33].

Chauvet and Collier [38] observe that aid can be an effective component for building state capacity both directly and indirectly. Strategies to reverse fragility and poor health outcomes however need to be purposeful, directed at multiple layers of state and society and long-term. They need to address the different market segments and the different market functions of supply and demand, rules and supporting functions and recognise that the provision of healthcare is determined by market considerations. It also requires sustained effort to develop the capacity of the state in budgeting, prioritising policy action and resources, coordination of related activities, monitoring, improved regulatory environment, analysis and delivery of tangible health outcomes. It includes working with the state to define the roles and responsibilities of the public sector in stewardship with aid flows aligned to country needs rather than donor country strategic needs [5].

Donors also need to use their influence to ensure that the non-state actors it funds engage in a meaningful way with the state rather than simply playing lip service to engagement. Donors funding health activities, for example, are in a position to demand evidence that agencies that they fund have engaged with the state, discussed their proposals with the MSPP before awarding contracts. As well as top-down strategies, bottom up strategies are essential [18]. This includes working with local village and health committees and looking at how the design of a public health program can provide opportunities and incentives for collective action to demand better services [18, 33]. Other strategies include increasing demand-side awareness, understanding what incentives would encourage self-regulation and the formation of voluntary professional bodies to create public authority outside of the state [5].

As with all research, our research has limitations. Most importantly, the data available in the scientific and “grey” literature primarily refers to the public sector; yet as the case study has demonstrated there is a thriving health market beyond the public sector. There is a need for an in-depth analysis that extends our understanding of the public health sector to a comprehensive representation of all contributors to the health system as a whole, mapping the supply and demand factors operating within the formal, informal, private, non-governmental organization and faith-based market segments for Haïti. Secondly, while the approaches used in this study have allowed the collection of rich, qualitative information, the study was also constrained by this unevenness of the available information, with its bias towards the state sector and to a lesser extent, not-for-profit private providers, resulting in uneven insights in relation to other important, but poorly documented, market segments.

## Conclusion

Characteristics of the health system described here, such as reduced public provision health, weak regulation, market diversification and the commodification of public goods are seen elsewhere. This case study suggests that donors need to develop new ways of working in fragile and conflict affected environments, recognizing the need to accept and work with this blurring of traditional mutually-exclusive categories. This broader understanding includes recognising that that effective, accountable formal institutions evolve through a complex interaction between state and society, public and private actors, formal and informal institutions [18]. The new paradigms of operation will require working with the state, as well as exploring ways of harnessing the health service provision that occurs beyond the reach of the state, documenting this process so that it supplements current, but poorly explored health systems research. This is the next research and policy challenge: to explore the complex structures of health service provision in these under-governed areas, to identify within them the existing or emergent systems of governance and accountability, and to work with them to bridge the gaps in health care provision, to ensure that top-down capacity building initiatives, when they eventually reach these marginal areas, engage an effective civil society response.
